# Notalgia Paresthetica as a differential diagnosis of chronic itching: report of two cases

**DOI:** 10.1186/1939-4551-8-S1-A242

**Published:** 2015-04-08

**Authors:** Catarina Furlan, Cintia Bassani, Lorena Petry, Mariana Monteiro, Romero Kopke, Maria Elisa Andrade, Wilson Aun, João Ferreira Mello

**Affiliations:** 1 Hospital Servidor Público Estadual De São Paulo, Brazil

## Background

Notalgia Paresthetica is part of the differential diagnosis of chronic itching. For years it was confused with one of the clinical varieties of cutaneous amyloidosis, being called "dorsal amyloidosus lichen", however, it was observed that the deposition of amyloid substance is not the cause, but the consequence of pruritus. It is characterized by itching and hyperpigmentation, usually in the scapular region. Symptoms appears in the areas of T2 to T6 dermatomes and affect more frequently females above 50 years. The pathophysiology involves the anatomical path of the spinal nerves, as well as neuropeptides released by nerve fibers in the peripheral nervous system. Some works relates it to the changes in the spine (compressions, hernias and trauma). The diagnosis is clinical with complementary examinations, such as CT and MRI. There is not a specific treatment, requiring a multidisciplinary approach.

## Methods

Report of two cases of notalgia paresthetica as a differential diagnosis of chronic itching.

## Results

In 2013 we had two female patients, 43 and 48 years old, both with hyperchromic brownish spots on the dorsal region and one also with supra umbilical lesion. Both without continuous use of medications. One of them complaining of recurrent candidiasis for 5 years, which was investigated revealing IgA deficiency associated. For the differential diagnosis of chronic itching, after detailed anamnesis, laboratory tests and imaging studies were requested:

- Patch test: weak reactor for Potassium Dichromate and Ammonium Thioglycolate (48h/96h: +/+) in only one of the patients.

- Prick test: negative.

- One of them performed MRI with paramedial disc protrusion to the left between C4 and C5, diffuse disc bulging rectifying the ventral dural sac between C5-C6, and disc protrusion compressing the ventral dural sac between C7-T1. The other patient underwent CT, with marginal osteophytes in the vertebral bodies, Schmörl nodes in vertebral plateaus of D8 and D11.

## Conclusions

Notalgia Paresthetica is usually an underdiagnosed disease, so, most professionals treat only the symptoms. The correct diagnosis is necessary for appropriate treatment.

